# Developmental *MYH3* Myopathy Associated with Expression of Mutant Protein and Reduced Expression Levels of Embryonic MyHC

**DOI:** 10.1371/journal.pone.0142094

**Published:** 2015-11-06

**Authors:** Malgorzata Pokrzywa, Michaela Norum, Johan Lengqvist, Mehrnaz Ghobadpour, Saba Abdul-Hussein, Ali-Reza Moslemi, Homa Tajsharghi

**Affiliations:** 1 Department of Clinical and Medical Genetics, University of Gothenburg, Gothenburg, Sweden; 2 Proteomic Core Facility, Sahlgrenska Academy, University of Gothenburg, Gothenburg, Sweden; 3 Department of Pathology, Institute of Biomedicine, Sahlgrenska Academy, University of Gothenburg, Gothenburg, Sweden; 4 Systems Biology Research Centre, School of Biomedicine, University of Skövde, Skövde, Sweden; Institut de Myologie, FRANCE

## Abstract

**Objective:**

An essential role for embryonic MyHC in foetal development has been found from its association with distal arthrogryposis syndromes, a heterogeneous group of disorders characterised by congenital contractions. The latter probably result from severe myopathy during foetal development. Lack of embryonic muscle biopsy material and suitable animal models has hindered study of the pathomechanisms linking mutations in *MYH3* to prenatal myopathy.

**Methods and Results:**

We determined the pathomechanisms of developmental myopathy caused by recurrent p.Thr178Ile *MYH3* heterozygosity, using patient-derived skeletal muscle cells in culture as an experimental disease model to emulate early embryonic development. These cultured cells were processed for discrimination and quantitative analysis of mutant and wild-type *MYH3* alleles and MyHC transcripts, real-time RT-qPCR, sequence analysis, immunofluorescence microscopy, immunoblot, and proteomic assessments. Involvement of the ubiquitin proteasome system was investigated in patients with p.Thr178Ile mutations in *MYH3* and *MYH2*. We found equal overall expression of mutant and wild-type MyHC mRNAs and proteins. Compared to the controls, however, expression of embryonic MyHC transcripts and proteins was reduced whereas expression of myosin-specific E3 ubiquitin ligase (MuRF1) was increased. We also found delayed myofibrillogenesis and atrophic myotubes but structured sarcomeres.

**Conclusion:**

In conclusion, this study suggests that developmental p.Thr178Ile *MYH3* myopathy is associated with a combined pathomechanism of insufficient dosage of functional embryonic MyHC and production of mutant protein.

## Introduction

Myosin heavy chain (MyHC) is the main component of sarcomeric thick filaments. It converts chemical energy from ATP hydrolysis into mechanical force for muscle contraction [1]. In humans, several isoforms of striated muscle MyHC have been described. They are encoded by different genes, which are expressed and regulated in a tissue- and developmental- specific manner [2–6]. There are three major MyHC isoforms in skeletal muscle of adult human limb: MyHC I, also called slow/β-cardiac MyHC, is encoded by *MYH7* and is expressed in slow, type-1 muscle fibres and in the ventricles of the heart; MyHC IIa (from *MYH2*) is expressed in fast, type-2A muscle fibres; and MyHC IIx (from *MYH1*) is expressed in fast, type-2B muscle fibres [7]. In addition, there are two MyHC isoforms expressed during foetal development in humans: embryonic MyHC, encoded by *MYH3*, and perinatal MyHC, encoded by *MYH8*. The expression of these isoforms declines rapidly after birth and they are not normally expressed in adult human limbs, except during muscle regeneration [8, 9].

Mutations in MyHC genes are an important cause of various myopathies [10]. The identification of dominant *MYH3* and *MYH8* mutations associated with distal arthrogryposis (DA) syndromes has recently highlighted the essential roles of the embryonic and perinatal MyHC isoforms for normal foetal development. Different mutations spanning the *MYH3* coding region have been associated with Freeman-Sheldon syndrome (FSS), the most severe form of DA, and Sheldon-Hall syndrome (SHS), the milder and most common form of DA. At least 18 missense mutations located in the globular myosin head domain, potentially affecting the binding sites for actin or nucleotides, and rod domain of embryonic MyHC have been associated with DA1, DA2A (FSS), and DA2B (SHS) [11–13].

The recurrent *MYH3* mutation p.Thr178Ile has been identified in several cases as a cause of FSS and SHS with dominant mode of inheritance, showing the deleterious effect of this mutation on the function of embryonic MyHC [11, 12]. Muscle biopsy specimens from one 15-month-old patient who carried the p.Thr178Ile mutation showed predominance of type-1 fibres and numerous fibres expressing the perinatal isoform of MyHC (*MYH8*). However, analysis at the RNA and protein levels indicated that embryonic MyHC was not expressed in any of the muscle biopsy specimens (12). The evidence for mild and variable myopathic features postnatally and the pathological up-regulation of the perinatal MyHC isoform in this patient suggested that there is severe myopathy during foetal development that causes joint contractures, leaving residual defects in muscle when expression of the embryonic MyHC is down-regulated [11]. Interestingly, the p.Thr178Ile mutation in *MYH3* has recently been identified in its paralogue in MyHC IIa (Thr178Ile from *MYH2*) as a cause of myosin IIa myopathy with a recessive mode of inheritance, demonstrating the deleterious effect of the p.Thr178Ile mutation on myosin [14]. Muscle biopsies from the patient showed no expression of *MYH2* transcript and no expression of the corresponding MyHC IIa protein.

Due to the lack of muscle biopsy material from embryos and the lack of suitable animal models, it is not yet known how *MYH3* mutations cause MyHC dysfunction during foetal development. We studied the expression patterns of wild-type and mutated embryonic MyHC at the RNA and protein levels in regenerating skeletal muscle cells from a patient with the p.Thr178Ile mutation in the *MYH3*. We found reduced expression of embryonic MyHC transcripts associated with lower amounts of embryonic MyHC protein, together with increased expression of myosin-specific E3 ubiquitin ligase MuRF1. This may indicate the involvement of the ubiquitin proteasome system (UPS), probably by transcriptional regulation of MyHC.

## Materials and Methods

### Analysis of muscle biopsy and tissue cultures

This study has been reviewed and approved by the ethical review committee in the Gothenburg region. After obtaining written informed consent, an open muscle biopsy specimen was obtained from the quadriceps and tibialis anterior muscles of patients with p.Thr178Ile mutations in *MYH2* or *MYH3*. Myoblasts from the patient with the p.Thr178Ile mutation in *MYH3* were isolated from the skeletal muscle tissue and cultured, as previously described [15]. Standardised human myoblast batches from five donors with no clinical signs of muscle disease were provided by MYOSIX through a collaborative program with Association Francaise contre les Myopathies (AFM); these were used as controls. Three control subjects were age and sex-matched with the patient. Isolated cells, stored in liquid nitrogen at the second passage, were cultured and allowed to differentiate for 1, 2, 3, 6, 8, 10, 16, 24, 32, and 49 days (D1‒49 differentiated myotubes) as previously described [16]. Cultured cells were processed for quantitative analysis of transcripts, sequence analysis, immunofluorescence, immunoblot, and proteomic assessments. Skeletal muscle tissue from a patient with recessive myosin IIa myopathy associated with the p.Thr178Ile mutation in *MYH2* [14] was processed for immunoblot analysis. Details of the methods are provided in S1 File in the Supporting information posted online.

### Statistical analysis

Statistical evaluations were performed with IBM SPSS Statistics 20 for Windows (IBM Corporation, Armonk, NY) using an independent-samples *t*-test and one-way ANOVA.

## Results

### Equal expression of mutant and wild-type MYH3 transcripts in the cultured, differentiated myotubes

**Fig 1:**
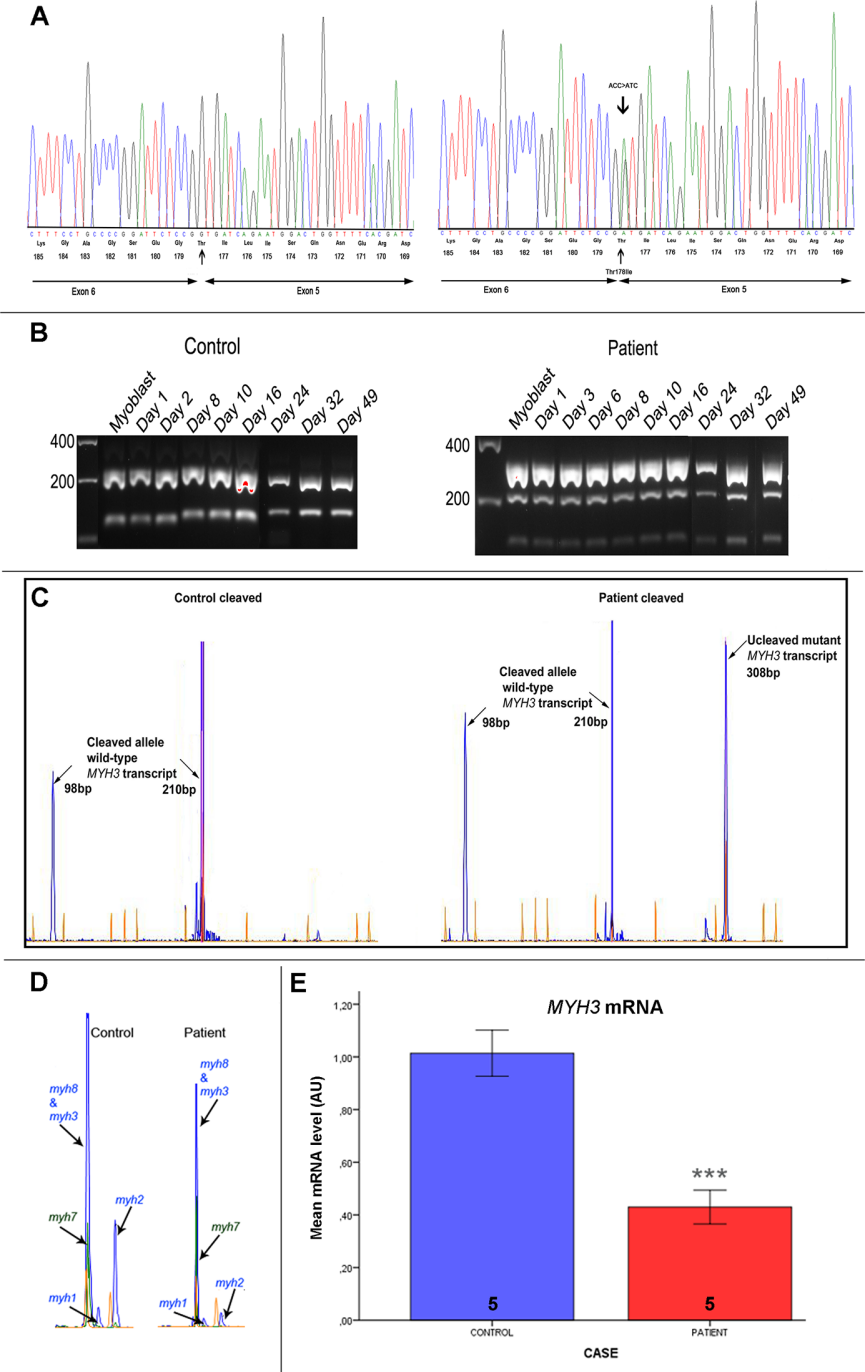
Mutation determination and quantitative analysis of RNA. (A) Sequence chromatograms of the entire exon 5 and part of exon 6 of *MYH3* in the control (left panel) and the patient who was heterozygous for the *MYH3* mutation p.Thr178Ile (c.602C>T), which changes threonine at position 178 of MyHC to isoleucine. The p.Thr178Ile mutation is shown in the reverse orientation. (B) Analysis of PCR-amplified and *BsawI*-treated fragments derived from *MYH3* alleles in differentiating myoblasts from a control (left panel) and from the patient (right panel), using 2% agarose. Treatment of an amplified 308-bp fragment of the wild-type *MYH3* allele with *BsaWI* generated 210-bp and 98-bp fragments, whereas the c.602C>T mutation eliminated the cleavage site in the mutated *MYH3* allele. (C) Illustration of quantitative analysis of the relative expression of mutated and normal *MYH3* alleles in myoblasts and differentiating skeletal myoblasts from control and patient. This was based on treatment of reverse transcriptase (RT)-PCR-amplified *MYH3*-derived fragments with *BsaWI*. The fluorescent PCR products were separated in polyacrylamide gels and the intensities of the respective peaks were analysed. (D) Illustration of quantitative analysis of the relative expression of myosin heavy chain (MyHC) I (*MYH7*), MyHC IIa (*MYH2*), MyHC IIx (*MYH1*), embryonic MyHC (*MYH3*) and perinatal MyHC (*MYH8*) transcripts in differentiating skeletal myoblasts from control and patient, based on RT-PCR. (E) Levels of *MYH3* mRNA determined by RT-qPCR with specific Taqman probes and normalised to GAPDH mRNA. The levels of *MYH3* transcripts from cultured cells (D3, D6, D8, D10, and D15) derived from the patient were compared to those derived from five controls, taken on corresponding days. The number of experiments/differentiated myotubes is indicated in the bars. Bars represent mean ± SD. ***p < 0.001(significant difference compared with controls, one-way ANOVA).

Embryonic MyHC is the main isoform of MyHC expressed during muscle regeneration, which can readily be reached in differentiated cultured muscle cells. Thus, complementary DNA (cDNA) prepared from cultured muscle cells of the patient carrying the p.Thr178Ile mutation was used in order to examine the expression of this *MYH3* mutation. The appearance of the mutated allele was confirmed by sequence analysis (Fig 1A). In addition, sequence analysis of a cDNA fragment covering exon 5 and exon 6 showed that the c.602C>T mutation, located at the last nucleotide of exon 5, did not affect proper splicing of exon 5 in the *MYH3* transcript, as illustrated in Fig 1A.

Consistent with the results from the sequence analysis, restriction fragment length polymorphism (RFLP) analysis showed that the mutated allele was present in the patient at the mRNA level. The missense mutation c.602C>T in *MYH3* eliminated a restriction site for endonuclease *BsaWI* (Fig 1B). Cleavage of the amplified fragment of cDNA, prepared from cultured muscle cells of the patient, resulted in fragments of 308 bp (mutant allele), 210 bp, and 98 bp (wild-type allele). Quantification of mutant and wild-type *MYH3* transcripts by RFLP fragment analysis indicated that both alleles were expressed more or less equally at the mRNA level in cultured muscle cells from the patient (the mutated allele represented 50–55% of the total). In the control cultured muscle cells, the presence of wild-type *MYH3* transcripts alone was confirmed (Fig 1C).

### Reduced expression levels of MYH3 transcript in the cultured, differentiated myotubes

Detailed analysis of the relative expression of different MyHC mRNAs (from *MYH3*, *MYH8*, *MYH7*, *MYH2*, *MYH4*) was done by reverse transcriptase polymerase chain reaction (RT-PCR). Analysis of the differentiated myotubes from the patient showed 35% lower expression levels of *MYH3* and *MYH8* together when compared with controls from the corresponding days. Interestingly, after one week of culture in differentiation medium, control myotubes showed an increase in the relative amount of adult *MYH2* transcript whereas patient myotubes at the same time point had undetectable levels of this isoform (Fig 1D). Correspondingly, the result from the quantitative analysis indicated that the relative expression level of *MYH7* transcript was 45% higher in the patient material than in the controls. Quantitative analysis to discriminate the expression of embryonic and perinatal MyHC isoforms revealed 42% lower expression of *MYH3* transcript in the myotubes from the patient, relative to the controls from corresponding days. Furthermore, real-time RT-qPCR confirmed that there were significantly lower levels of *MYH3* transcript in cultured myotubes from the patient who was heterozygous for the p.Thr178Ile mutation in *MYH3* than in those from controls (Fig 1E).

### Reduced expression of embryonic MyHC in cultured myotubes

**Fig 2:**
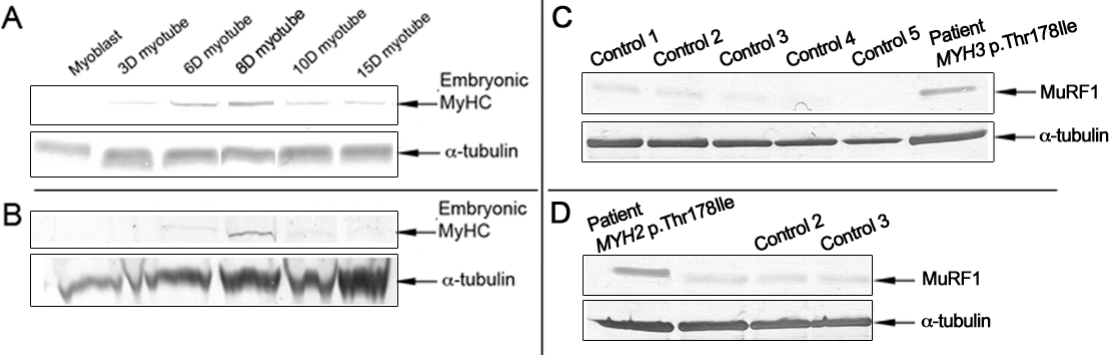
Immunoblot analysis of the embryonic isoform of MyHC in cultured cells and investigation of the levels of expression of MuRF1 in cultured cells and skeletal muscle. (A and B) Representative immunostaining with the antibody to embryonic MyHC in western blot material (myoblasts and differentiated myotubes) from a control individual (A) and from the patient with the p.Thr178Ile mutation in *MYH3* (B). Embryonic MyHC expression was at a lower level in the patient material than in the control material taken on corresponding days. (C and D) Representative western blots stained with anti-MuRF1. Protein from D8 differentiated cultured cells (with highest expression levels of embryonic MyHC) derived from the patient with the p.Thr178Ile *MYH3* mutation and from five controls (C), and proteins from skeletal muscle tissues from the patient with the p.Thr178Ile *MYH2* mutation and from three controls (D). MuRF1 expression was higher in the patients with p.Thr178Ile mutations than in the controls. We used α-tubulin as a loading control.

The levels of MyHC proteins in general and that of embryonic MyHC in particular were assessed. Initially, the total protein levels of MyHC isoforms extracted from cultured differentiated myotubes of the controls and patient were assessed using SDS-PAGE. There were 40–45% lower levels of total MyHCs in the patient relative to the controls from corresponding days (data not shown). We then investigated the expression of embryonic MyHC by immunoblotting of proteins extracted from cultured differentiated myotubes from the patient and the controls (Fig 2A and 2B). Chemiluminescence analysis indicated the presence of 220‒kDa embryonic MyHC in differentiated myotubes from the controls and the patient (day 1 to day 16). The expression increased from day 1 to day 8 but declined from day 8 to day 16. However, markedly lower expression of embryonic MyHC was detected in differentiated myotubes from the patient relative to the controls from corresponding days. Immunoblotting with the anti-perinatal MyHC antibody did not work under our experimental conditions.

### Involvement of the ubiquitin proteasome system (UPS)

Considering that MuRF1 is an striated muscle-specific E3 ubiquitin ligase that mediates ubiquitination of sarcomeric proteins, including MyHC, for degradation by the UPS [17, 18], we speculated that the expression levels of MuRF1 would be up-regulated in patients with *MYH3* mutation p.Thr178Ile or *MYH2* mutation p.Thr178Ile. Indeed, there was an roughly 3-fold increased expression of MuRF1 in protein extract from cultured myotubes from the patient with reduced MyHCs in general and embryonic MyHC in particular, relative to the controls (Fig 2C and S1A Fig). Similarly, immunoblot analysis of skeletal muscle tissue from the patient with recessive myosin IIa myopathy, associated with the *MYH2* mutation p.Thr178Ile and total absence of MyHC IIa, revealed a roughly 4-fold increased expression of MuRF1 relative to the controls (Fig 2D and S1B Fig). Together, these observations might suggest that down-regulation of embryonic MyHC or MyHC IIa is mediated by increased expression of MuRF1.

### Equal expression of mutant and wild-type embryonic MyHC at the protein level

To specifically determine whether mutant embryonic MyHC (p.Thr178Ile) was expressed in cultured myotubes from the patient, we used a targeted proteomic analysis. To ensure optimal sensitivity and specificity, the full 140 K resolution of the Q Exactive instrument was used to collect fragment and full MS data for wild-type (ENQSILITGESGAGK) and mutant (ENQSILIIGESGAGK) embryonic MyHC tryptic peptides covering the p.Thr178 site.

**Fig 3:**
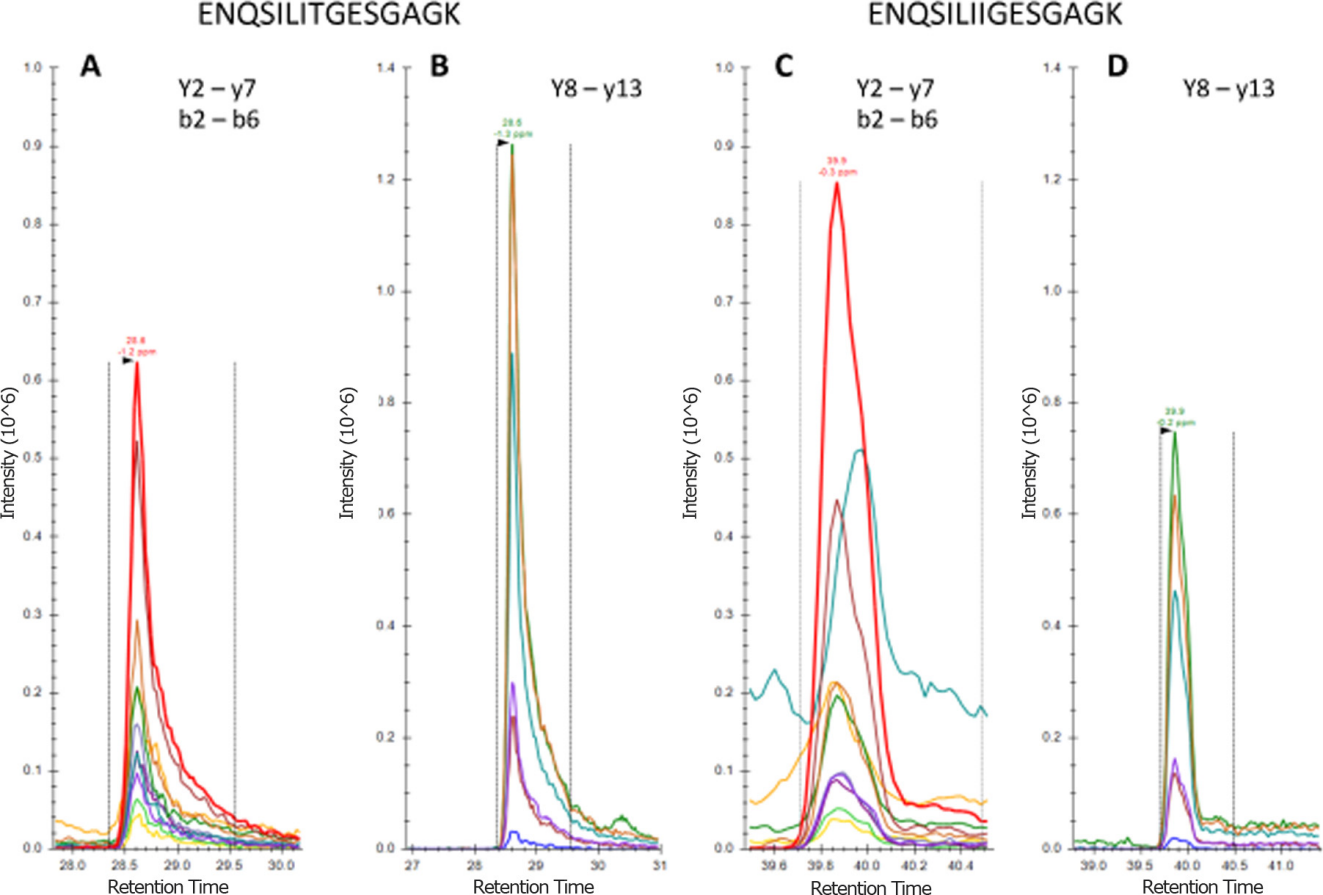
Detection and relative quantification of wild-type and mutated embryonic MyHC. (A and B) Extracted ion chromatograms (XICs) for wild-type and (C and D) mutant embryonic MyHC tryptic peptides eluting at 28.6 and 39.9 minutes are shown as indicated. Note that panels A and C have been adjusted to equal intensity, as have panels B and D.

At retention times of 28.6 and 39.9 minutes, common (y2-y7 and b2-b6) and sequence- specific (y8-y13) fragment ions were observed for wild-type (Fig 3A and 3C) and mutant peptides (Fig 3B and 3D), respectively. Peptide identification was apparent by high precursor (< 2 ppm, S2 and S3 Figs) and fragment mass accuracy (RMS < 1.8 ppm for 12 (wild-type) and 13 (mutant) y-ion fragments, S4 and S5 Figs). Importantly, the p.Thr178Ile substitution was apparent from six unique fragment ions in each case. Measured relative intensities of common (non-unique) fragment ions indicated a 1:1 ratio of wild type to mutant (c.f. Fig 3A and 3C).

### Delayed myofribrillogenesis and myotube atrophy in cells cultured from the patient

Maintenance of muscle differentiation in the myocytes derived from a muscle biopsy of the patient was assessed. The myogenic potential of these cells was confirmed by staining for desmin. *In vitro* differentiation of these myoblasts and myotube formation were markedly reduced compared to control cells, indicating delayed myofribrillogenesis. This apparent difference was evident at 3 days of differentiation, with infrequent formation of myotubes in the patient, contrasting with the predominant formation of myotubes with the distinctive alternating band patterns of the sarcomere in controls. This was confirmed by calculation of fusion index, which was significantly higher for the control myotubes (*p* < 0.001, one-way ANOVA). The mean fusion index for the control myotubes was 44% at day 3 and 67% at day 6 of differentiation, whereas only 16% and 27% for the patient myotubes, respectively (S6A Fig). Detailed analysis of the nucleation size of the myotubes showed 100% 2–3 nucleated 3-day myotubes and sporadically 4–5 nucleated 6-day myotubes in the patient. The control myotubes showed higher fusion capacity and some myotubes containing up to 20 nuclei (S6B Fig). In addition, the myotubes in the patient were significantly thinner than the cells in the controls (*p* < 0.001, one-way ANOVA), as determined by measuring myotube diameter. The mean diameter of 6-day myotubes was 11.03 μm (± 6.44, number of myotubes (n) = 27) in the controls and 5.15 μm (± 2.73, n = 31) in the patient (S6C Fig).

### Formation of sarcomere structure in cells cultured from the patient and control

Following 3 and 6 days of differentiation, cultures of control and patient myotubes were analysed for sarcomere formation. Overall, the staining patterns for many sarcomeric proteins were quite similar in control and patient myotubes.

**Fig 4:**
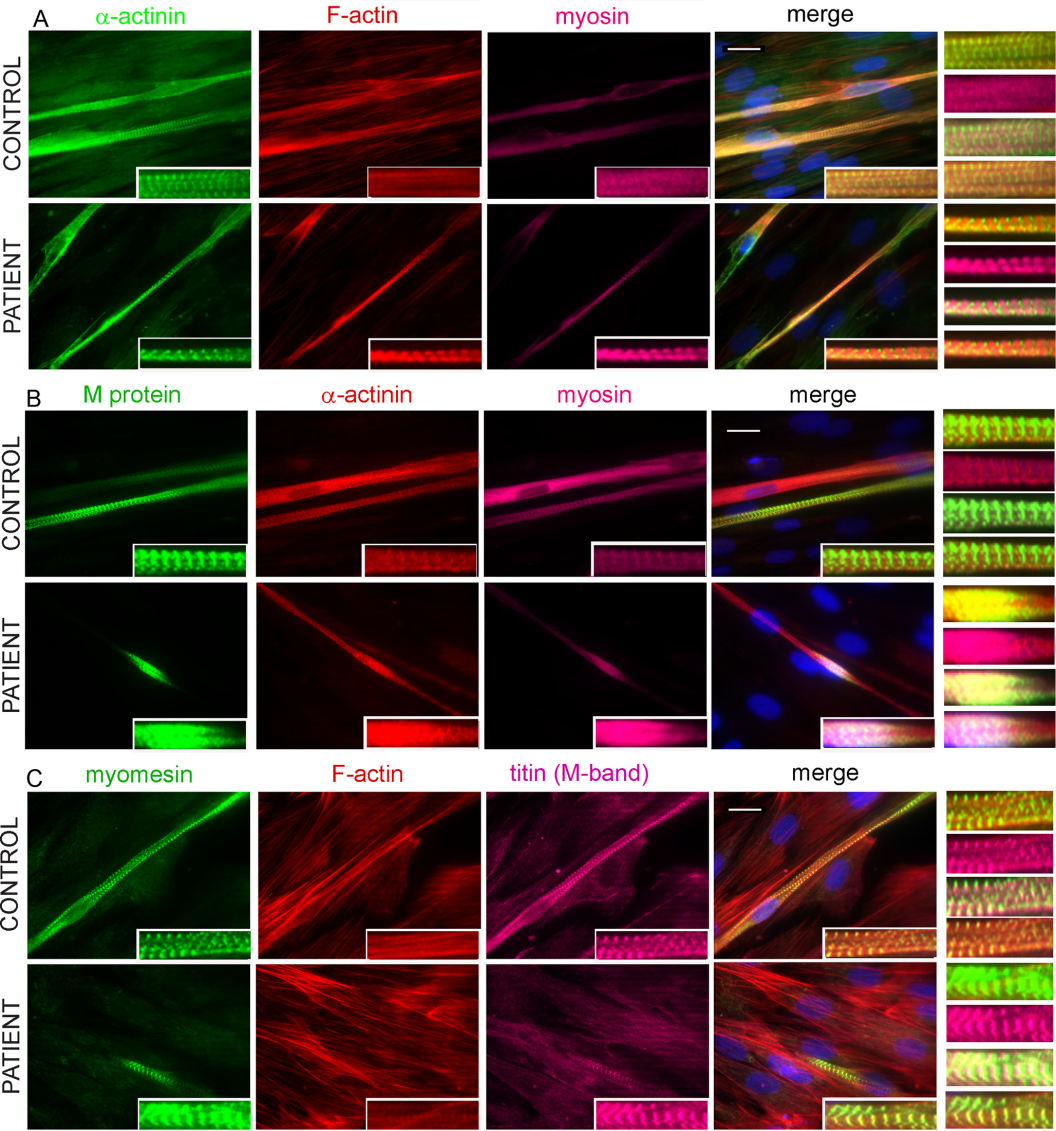
Formation of sarcomere structure of 6-day differentiated myotubes. (A) Triple staining was performed for α-actinin (green), F-actin (red), and myosin (magenta); (B) M-protein (green), F-actin (red), and myosin (magenta); (C) myomesin (green), F-actin (red), and M-band epitope of titin (magenta). The results were visualized with a Zeiss Axio Observer microscope (Carl Zeiss AG, Germany) at 63× magnification. All nuclei were counterstained with DAPI (blue). The repetitive well-structured sarcomere can be seen clearly in control myotubes and patient myotubes (insets). The bars represent 10 μm.

**Fig 5:**
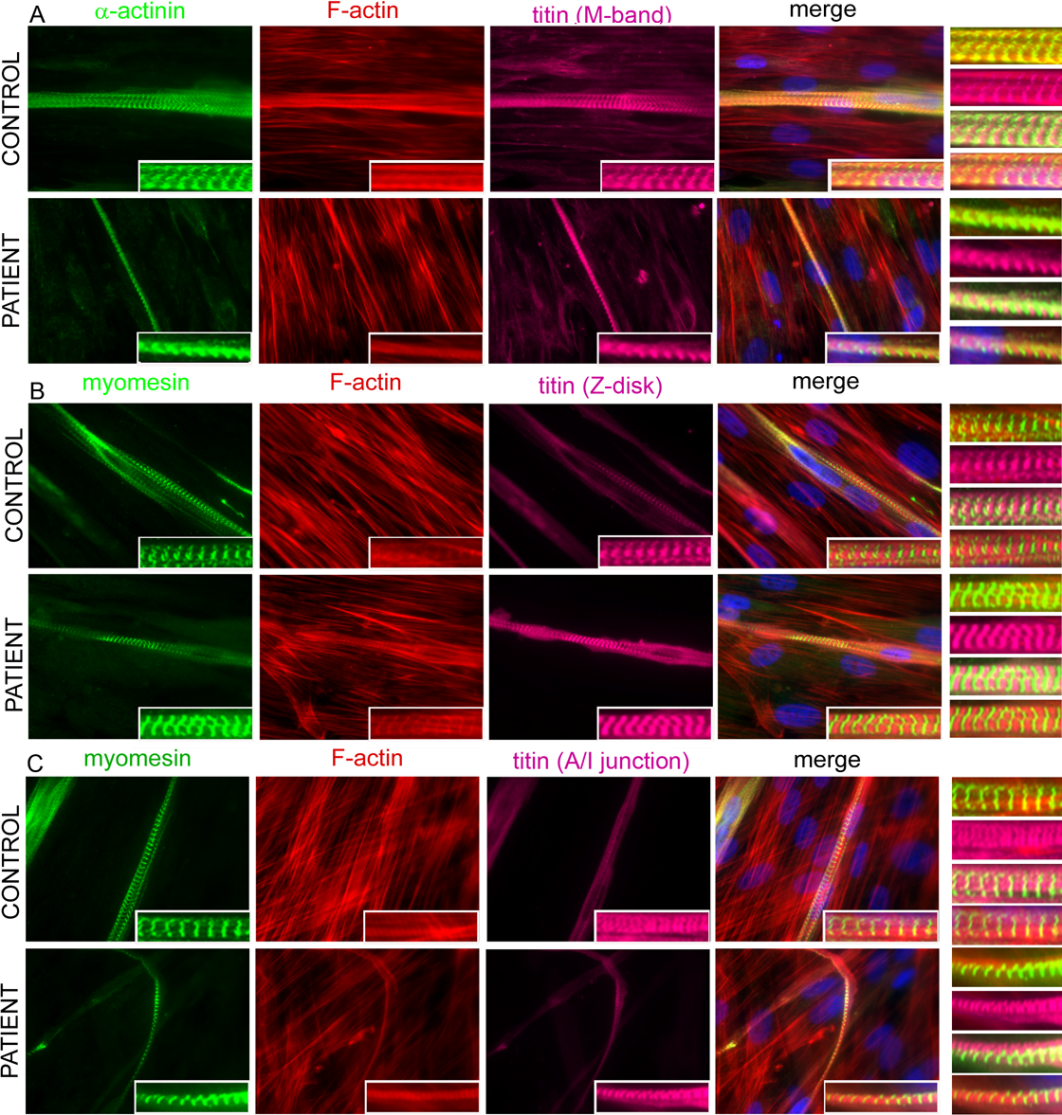
Co-immunofluorescence analysis of 6-day differentiated myotubes. (A) Triple staining was performed for α-actinin (green), F-actin (red) and M-band epitope of titin (magenta) (A); (B) myomesin (green), F-actin (red) and Z-disc epitope of titin (magenta); (C) myomesin (green), F-actin (red) and A/I junction epitope of titin (magenta). The results were visualised with a Zeiss Axio Observer microscope (Carl Zeiss AG, Germany) at 63× magnification. All nuclei were counterstained with DAPI (blue). The Z-disc can be seen clearly with α-actinin and Z-disc epitope of titin, and the M-band with myomesin, M-band epitope of titin (insets). Note the strikingly thinner myotubes in the patient compared to the cells in the control. The bars represent 10 μm.

We investigated the proper localisation of myosin at the A-band and the alternating band patterns of the sarcomere. Results from triple immunostaining for α-actinin, actin, and myosin or M-protein, α-actinin, and myosin indicated that there was no disruptive effect on sarcomere organisation and there was proper formation of A-band, M-band, and Z-disc in both control myotubes and patient myotubes (Fig 4A–4C). Results from further double and triple immunostaining indicated proper formation of M-band, where the myosin-containing thick filaments are anchored, in both control myotubes and patient myotubes (S7 Fig and Fig 5A–5C). Moreover, immunostaining for myomesin and M-band epitope of titin indicated their proper co-localisation at the M-band region of the sarcomere (Fig 4C). Triple-immunostaining for either α-actinin, actin, and M-band epitope of titin, or Z-disc epitope of titin, actin and myomesin additionally revealed the alternating staining patterns of Z-discs and M-bands in the patient and control 3-day myotubes (Fig 5A and 5B). The appearance of correctly alternating band patterns of the sarcomere was further demonstrated in 3- and 6-day myotubes of both patient and control by triple-immunostaining for myomesin, actin and A/I junction epitope of titin (Fig 5C).

### Expression patterns of embryonic and perinatal MyHC isoforms in myotubes

Finally, we investigated the expression of embryonic and perinatal MyHC isoforms at the protein levels in myotubes that had been differentiated for 3, 4, or 6 days. Consistent with the data from mRNA and immunoblot analyses, immunolocalisation studies of differentiated myotubes showed that the proportion of myotubes that were positively stained for embryonic MyHC was less in the patient material than in the control material. In contrast, the proportion of cells stained positively for perinatal MyHC in the myotube cultures derived from the patient was higher in myotubes derived from the controls (data not shown). Thus, there is reduced expression of embryonic MyHC and a relative increase in perinatal MyHC expression during early muscle development.

## Discussion

Embryonic MyHC is expressed during embryonic development [8, 9] and mutations in the gene encoding it (*MYH3*) cause a developmental myopathy [11, 12]. The Thr178Ile *MYH3* mutation has been reported several times in unrelated families with FSS and SHS, indicating that this amino acid position is a mutational hotspot [11] [12]. A correct understanding of the cause of the disease from *MYH3* mutation would require examination of muscle pathology during the developmental period when *MYH3* is expressed. However, due to lack of muscle biopsy material from embryos and of suitable embryo models in animals, it is not yet established how *MYH3* mutations cause structural and functional dysfunction during foetal development. To pinpoint the pathogenesis of developmental myopathy caused by *MYH3* mutation, we established primary cultures of muscle cells obtained from a patient with the p.Thr178Ile mutation in *MYH3* [11] as an experimental disease model. Skeletal muscle myoblasts, which are capable of proliferation and differentiation *in vitro*, emulate early embryonic development and muscle regeneration.

To date, the *MYH3* mutations identified to be associated with DA have shown a dominant mode of inheritance [11, 12]. Furthermore, most of the *MYH3* mutations are missense and would be expected to produce full-length mutant proteins, but it is not known whether the mutant allele is expressed. Thus, one could hypothesise that the mutated proteins affect muscle function during early development―either through insufficient expression level of *MYH3*, with insufficient dosage of a functional embryonic MyHC, or through a dominant negative effect of the mutated allele.

Since embryonic MyHC is the predominant MyHC isoform during early development [8, 9], the low amount of embryonic MyHC expression may not be tolerated, causing high developmental vulnerability and disease. Thus, the pathogenesis of a dominant *MYH3* mutation could result from insufficient amounts of functional embryonic MyHC. MyHC IIa, on the other hand, is one of three MyHC isoforms in adult human limb muscles and the other isoforms may to some extent compensate for a defect. The situation is therefore different in individuals with heterozygous *MYH2* null mutations, in whom the hemizygous loss of MyHC IIa is well tolerated [19]. The absence of MyHC IIa may support loss-of-function effects of the homozygous *MYH2* missense mutation p.Thr178Ile in patients with recessive myosin IIa myopathy [14]. This also ties in with clinically unaffected parents, since a loss-of-function effect of heterozygous p.Thr178Ile *MYH2* mutation is unlikely to contribute to disease in a haplo-insufficient manner [14]. However, the mechanisms by which the homozygous p.Thr178Ile *MYH2* missense mutation leads to a complete absence of MyHC IIa protein are obscure.

Taking together, the data from RNA, immunoblot, and immunolocalisation analyses of differentiated myotubes from the patient with p.Thr178Ile *MYH3* showed reduced expression of embryonic MyHC and a relative increase in perinatal MyHC expression. This probably reflects a developmental defect during early muscle development, through insufficient amount of embryonic MyHC. Furthermore, it can be concluded that the embryonic and perinatal isoforms of MyHC have unique structural and functional roles and are unable to substitute for one another. This is further supported by studies of mice that are null for different adult fast skeletal isoforms of MyHC. Although there was a relative compensatory increase in MyHC IIa and MyHC IId/x expression in the MyHC IId/x and MyHC IIb null mice, respectively [20], inactivation of the genes encoding MyHC IIb and IId/x has indicated that these genes are required for the normal development and functioning of adult skeletal muscle in the mouse, and they cannot be compensated for by each another [20–24].

Our results from transcription and immunoblot analyses showed a marked reduction of embryonic MyHC at the mRNA and protein levels in the patient with the p.Thr178Ile mutation in *MYH3*. Similarly, skeletal muscle tissue in the patient homozygous for *MYH2* p.Thr178Ile mutation, showed absence of MyHC IIa both at the mRNA level and at the protein level [14]. Knowing that the specificity of the ubiquitination of MyHC lies in the E3 ubiquitin ligase MuRF1, we assessed the involvement of MuRF1 in expression of the embryonic and IIa isoforms of MyHC associated with p.Thr178Ile mutations in *MYH3* and *MYH2*, respectively. We did indeed detect reduced expression of embryonic MyHC together with increased expression of MuRF1 in cultured myotubes of the *MYH3* patient. In addition, absence of MyHC IIa in the skeletal muscle tissue of the patient homozygous for p.Thr178Ile in *MYH2* was associated with increased expression of MuRF1. Together, these findings may suggest the involvement of UPS, and that MuRF1 may have a role in regulation of expression of MyHCs. It is likely that increased ubiquitination of MyHC mediated by MuRF1 for degradation via UPS leads to reduced levels of MyHC protein. An alternative mechanism would be that MuRF1 might indirectly reduce the level of MyHC protein by regulating the transcription of genes encoding MyHC. This is further supported by recent data demonstrating that over-expression of MuRF1 both in myocytes and in MuRF1-transgenic mice markedly reduced the levels of MyHC mRNAs, suggesting that MuRF1 degrades a MyHC-specific transcription factor [25].

The significantly reduced size of the myotubes in the patient with the p.The178Ile mutation in *MYH3* may indicate that heterozygosity for this *MYH3* mutation was sufficient to cause muscle atrophy during muscle development, and support the idea of a developmental myopathy. Given the fact that MuRF1 expression is up-regulated in atrophic muscle [26–29], the atrophic myotubes and the increased expression level of MuRF1 with decreased expression level of embryonic MyHC in cultured myotubes of the patient may further support the involvement of UPS in regulation of embryonic MyHC expression.

The results from quantitative analysis of transcripts demonstrated that there were equal levels of wild-type and mutant *MYH3* transcripts. Correspondingly, the presence of mutant p.The178Ile protein at a level similar to that of wild-type embryonic MyHC was revealed by advanced quantitative proteomic techniques. This indicates that mutant p.The178Ile embryonic MyHC protein is likely to be incorporated in the A-band of the sarcomere. Thus, another possibility would be that the *MYH3* mutation imposed dominant negative effects by incorporation of a functionally or structurally defective protein into sarcomeres. Delayed myofibrillogenesis may further indicate the impact of the p.Thr178Ile embryonic MyHC mutation in the formation of sarcomeric thick filaments during muscle development. However, the data from immunolocalisation analyses indicated the proper localisation of myosin at the A-band and M-band and the alternating band patterns of the sarcomere. These observations suggest that there was no structural defect in sarcomere formation by the p.Thr178Ile embryonic MyHC mutation. The p.Thr178Ile mutation is located adjacent to the highly conserved base of the ATP-binding pocket (Gly179-Thr186), so it may disrupt ATP binding or ATP hydrolysis. Thus, a functional rather than a structural defect is likely to be the consequence arising from such a mutation.

In conclusion, the Thr178Ile mutation in embryonic MyHC is associated with reduced expression levels of embryonic MyHC in cultured myotubes from the patient and that mutant protein is present in equal levels to the wild-type protein. Our data suggest that both mechanisms may have a role in the pathogenesis of DA associated with heterozygous *MYH3* mutation, which is probably accompanied by severe myopathy and muscle weakness during foetal development. In addition, our data may suggest that MyHC isogene expression in patients with p.Thr178Ile mutations in *MYH3* or *MYH2* is regulated by UPS and that MuRF1 may contribute to reduce levels of MyHCs, probably by transcriptional regulation of MyHC.

## Supporting Information

S1 Fig Quantification of MuRF1 expression levels in cultured cells and skeletal muscle. (A) The quantitative analysis of expression levels of MuRF1 detected in protein extract from differentiated myotubes from the patient with the p.Thr178Ile *MYH3* mutation relative to the controls from corresponding days, and (B) from skeletal muscle tissue from the patient with recessive myosin IIa myopathy, associated with the p.Thr178Ile *MYH2* mutation relative to the controls, normalized to α-tubulin. Levels of MuRF1 expression in controls are comparable. MuRF1 expression levels was increased 2,7-fold in the patient with the p.Thr178Ile *MYH3* mutation and 3,8-fold in the patient with the p.Thr178Ile *MYH2* mutation, relative to the controls.
(TIF)

S2 Fig Full MS (precursor) spectra for WT tryptic peptides covering the Thr178 position. Data was collected at 140 K resolution throughout.
(TIF)

S3 Fig Full MS (precursor) spectra for mutated tryptic peptides covering the Thr178Ile substitution. Data was collected at 140 K resolution throughout.
(TIF)

S4 Fig Multiplexed MS/MS spectra for WT tryptic peptides covering the Thr178 position. Data was collected at 140 K resolution throughout.
(TIF)

S5 Fig Multiplexed MS/MS spectra for mutated tryptic peptides covering the Thr178Ile substitution. Data was collected at 140 K resolution throughout.
(TIF)

S6 Fig Determination of the fusion index and diameter of myotubes. (A) Histogram represents the mean fusion index (%, ±SD) calculated for the control and patient myotubes at 3-day and 6-day of *in vitro* differentiation. (B) Histogram shows the frequency of nucleation size of the control and patient myotubes. Myotubes were divided into 3 size classes: myotubes with 2–3 nuclei, myotubes with 3–10 nuclei and myotubes with 10–30 nuclei. (C) Diagram shows mean myotube thickness (μm, ±SD) of control and patient 6-day differentiated myotubes.
(TIF)

S7 Fig Formation of myotube and sarcomere structure in differentiated myoblasts. (A) Double staining were performed with α-actinin (green) and M-band epitope of titin (red). (B) myomesin (green) and Z-disc epitope of titin. (C) α-actinin (green) and A-band epitope of titin. (D) myomesin (green) and A/I junction epitope of titin (red). The results were visualized in Zeiss Axio Observer microscope (Carl Zeiss AG, Germany) at 63x magnification. All nuclei were counterstained with DAPI (blue). The Z-disc can be seen clearly with α-actinin and Z-disc epitope of titin, the M-band can be seen with myomesin and M-band epitope of titin and A-band can be seen with A-band and A/I junction epitope of titin in the patient and a control 6-day differentiated myotubes (insets). Note the strikingly thinner myotubes in the patient compared to the cells in the control. The bars represent 10 μm.
(TIF)

S1 File Extended Materials and Methods, Results and References. (DOCX)
