# Supplementary figures and images for: Developmental MYH3 Myopathy Associated with Expression of Mutant Protein and Reduced Expression Levels of Embryonic MyHC
Source: PLoS One. 2015 Nov 6;10(11):e0142094. doi: 10.1371/journal.pone.0142094 (PMC4636365; doi:10.1371/journal.pone.0142094)

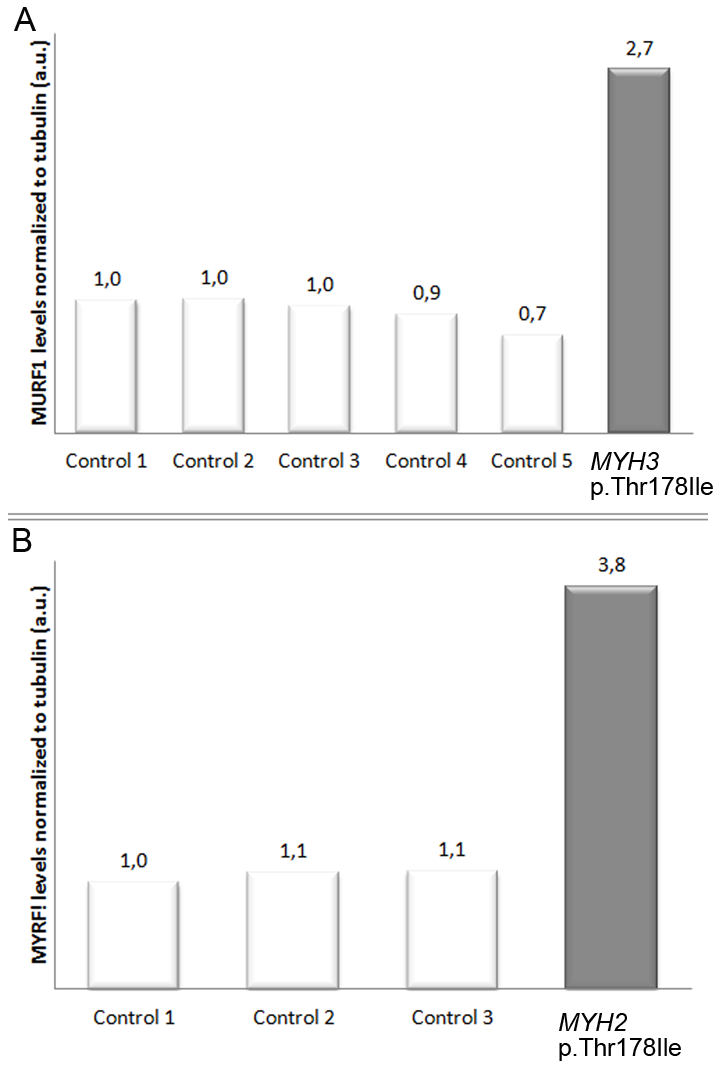

Supplement: S1 Fig — (A) The quantitative analysis of expression levels of MuRF1 detected in protein extract from differentiated myotubes from the patient with the p.Thr178Ile MYH3 mutation relative to the controls from corresponding days, and (B) from skeletal muscle tissue from the patient with recessive myosin IIa myopathy, associated with the p.Thr178Ile MYH2 mutation relative to the controls, normalized to α-tubulin. Levels of MuRF1 expression in controls are comparable. MuRF1 expression levels was increased 2,7-fold in the patient with the p.Thr178Ile MYH3 mutation and 3,8-fold in the patient with the p.Thr178Ile MYH2 mutation, relative to the controls. (TIF) [file pone.0142094.s001.tif]

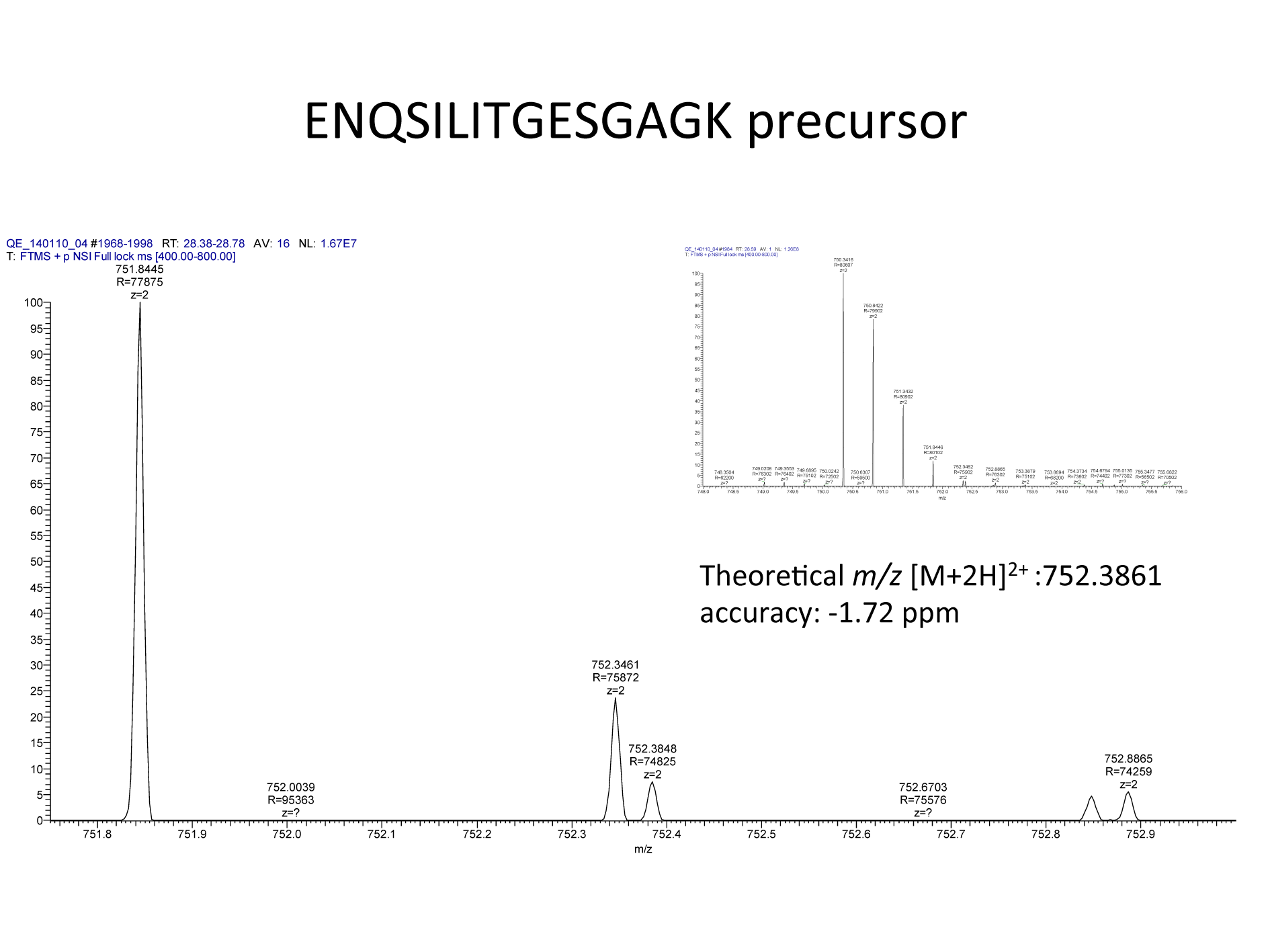

Supplement: S2 Fig — Data was collected at 140 K resolution throughout. (TIF) [file pone.0142094.s002.tif]

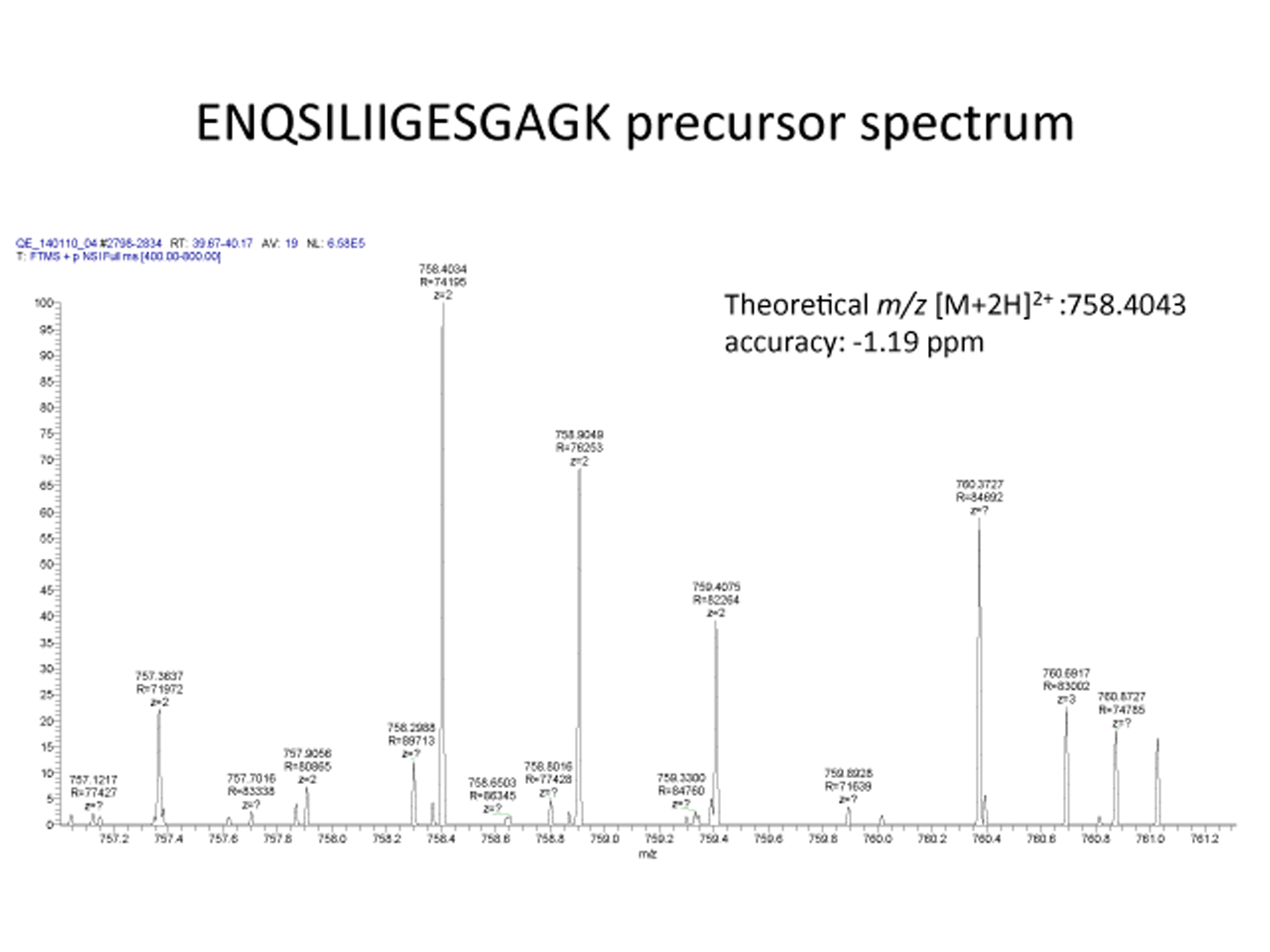

Supplement: S3 Fig — Data was collected at 140 K resolution throughout. (TIF) [file pone.0142094.s003.tif]

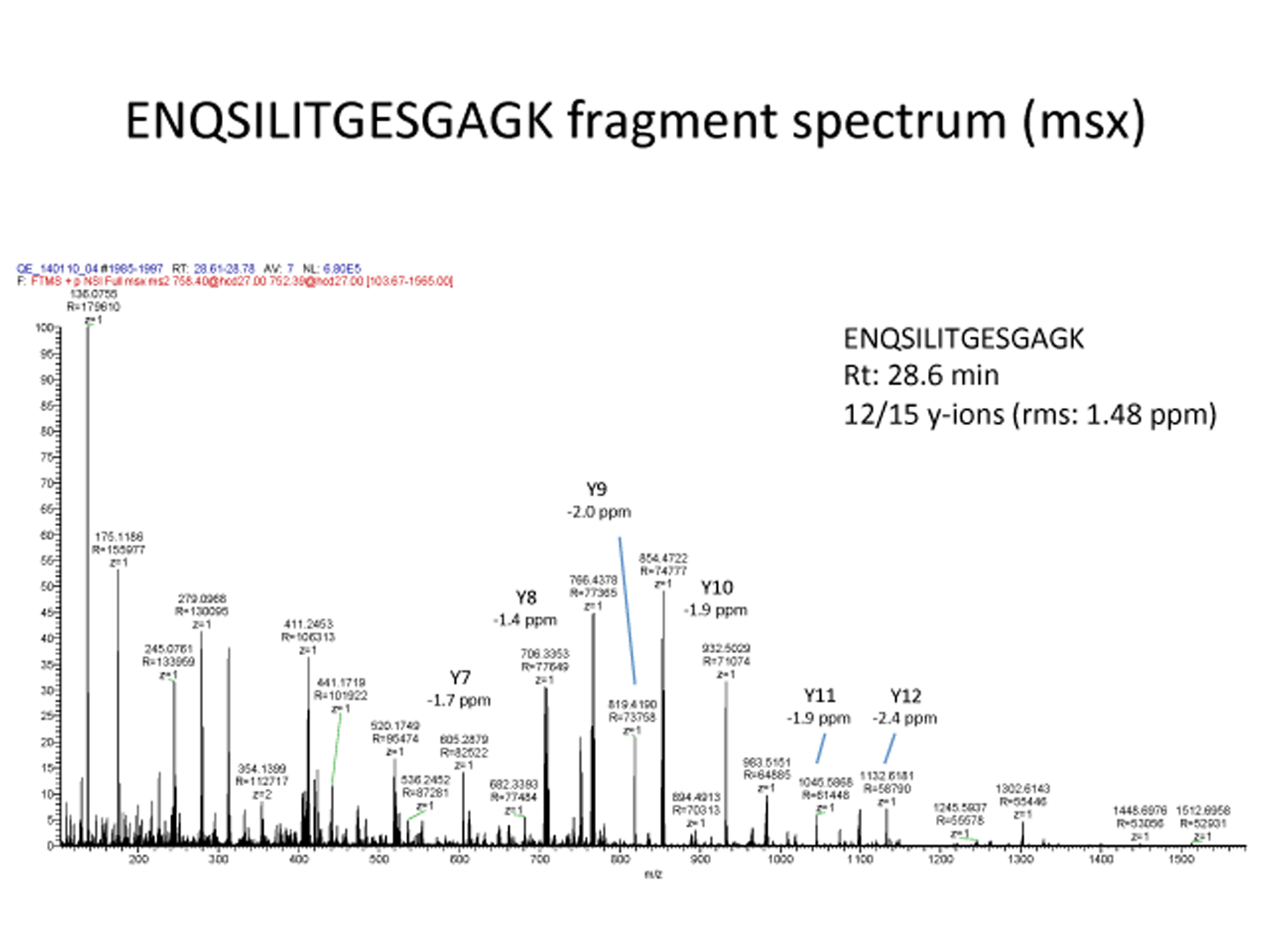

Supplement: S4 Fig — Data was collected at 140 K resolution throughout. (TIF) [file pone.0142094.s004.tif]

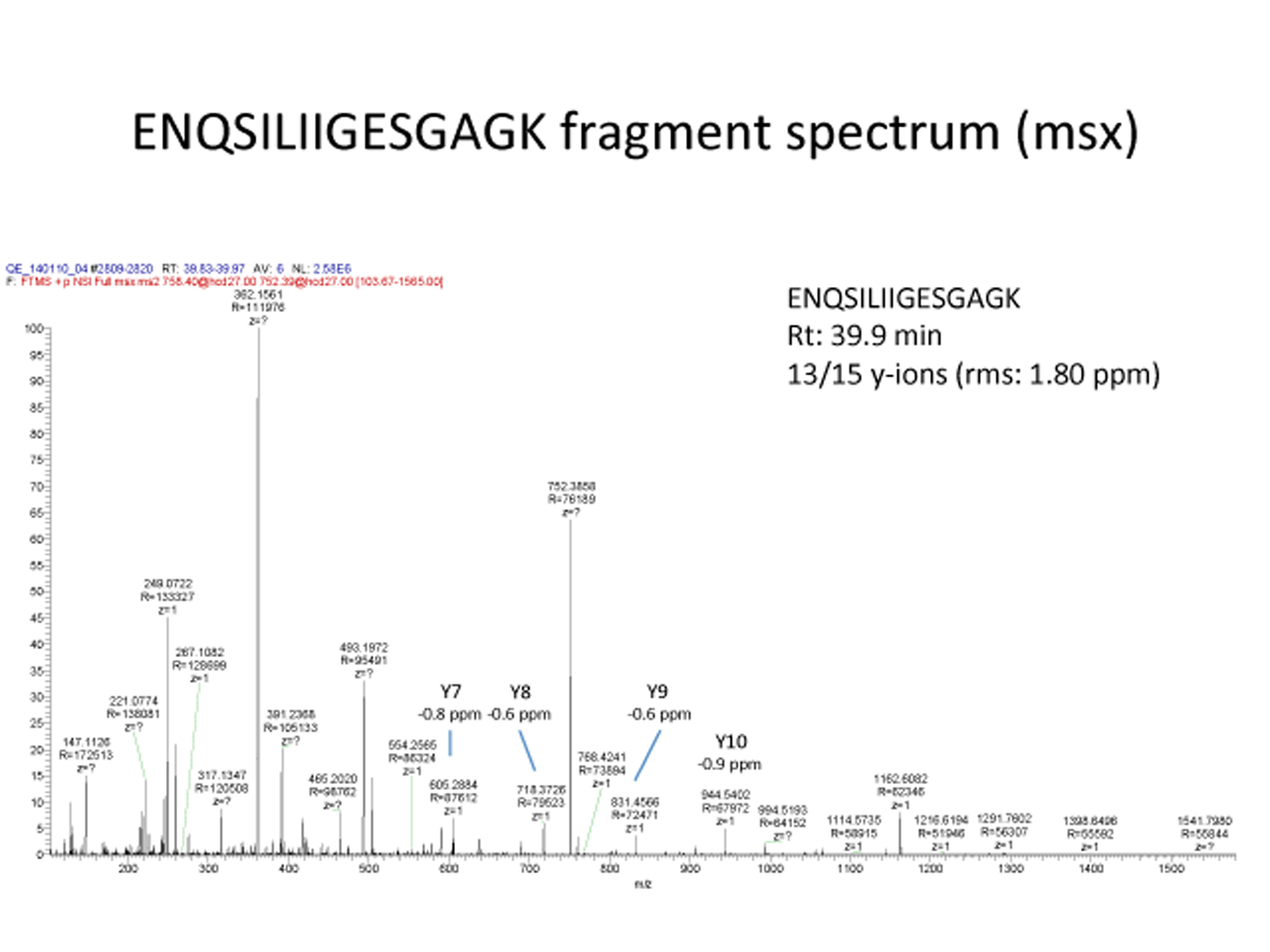

Supplement: S5 Fig — Data was collected at 140 K resolution throughout. (TIF) [file pone.0142094.s005.tif]

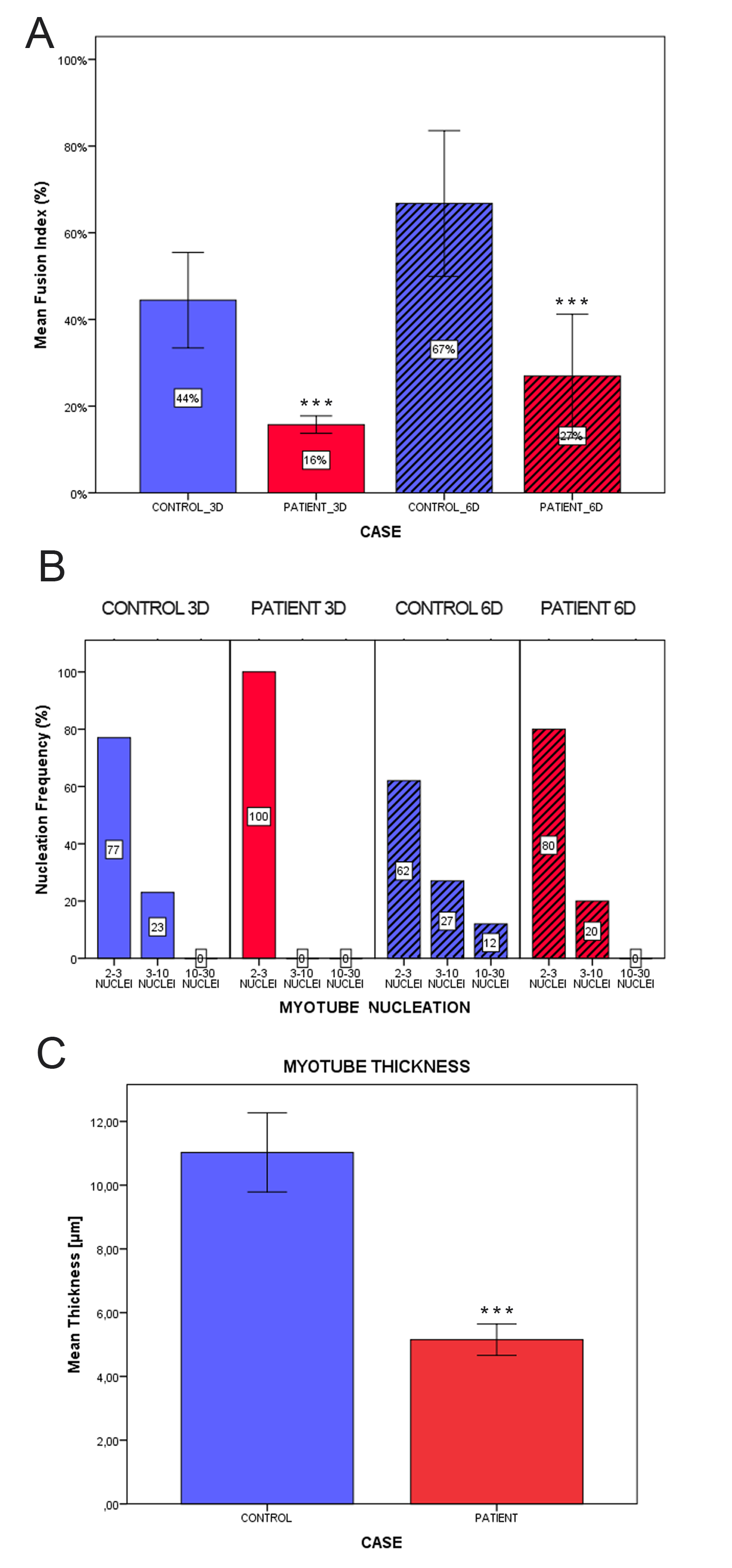

Supplement: S6 Fig — (A) Histogram represents the mean fusion index (%, ±SD) calculated for the control and patient myotubes at 3-day and 6-day of in vitro differentiation. (B) Histogram shows the frequency of nucleation size of the control and patient myotubes. Myotubes were divided into 3 size classes: myotubes with 2–3 nuclei, myotubes with 3–10 nuclei and myotubes with 10–30 nuclei. (C) Diagram shows mean myotube thickness (μm, ±SD) of control and patient 6-day differentiated myotubes. (TIF) [file pone.0142094.s006.tif]

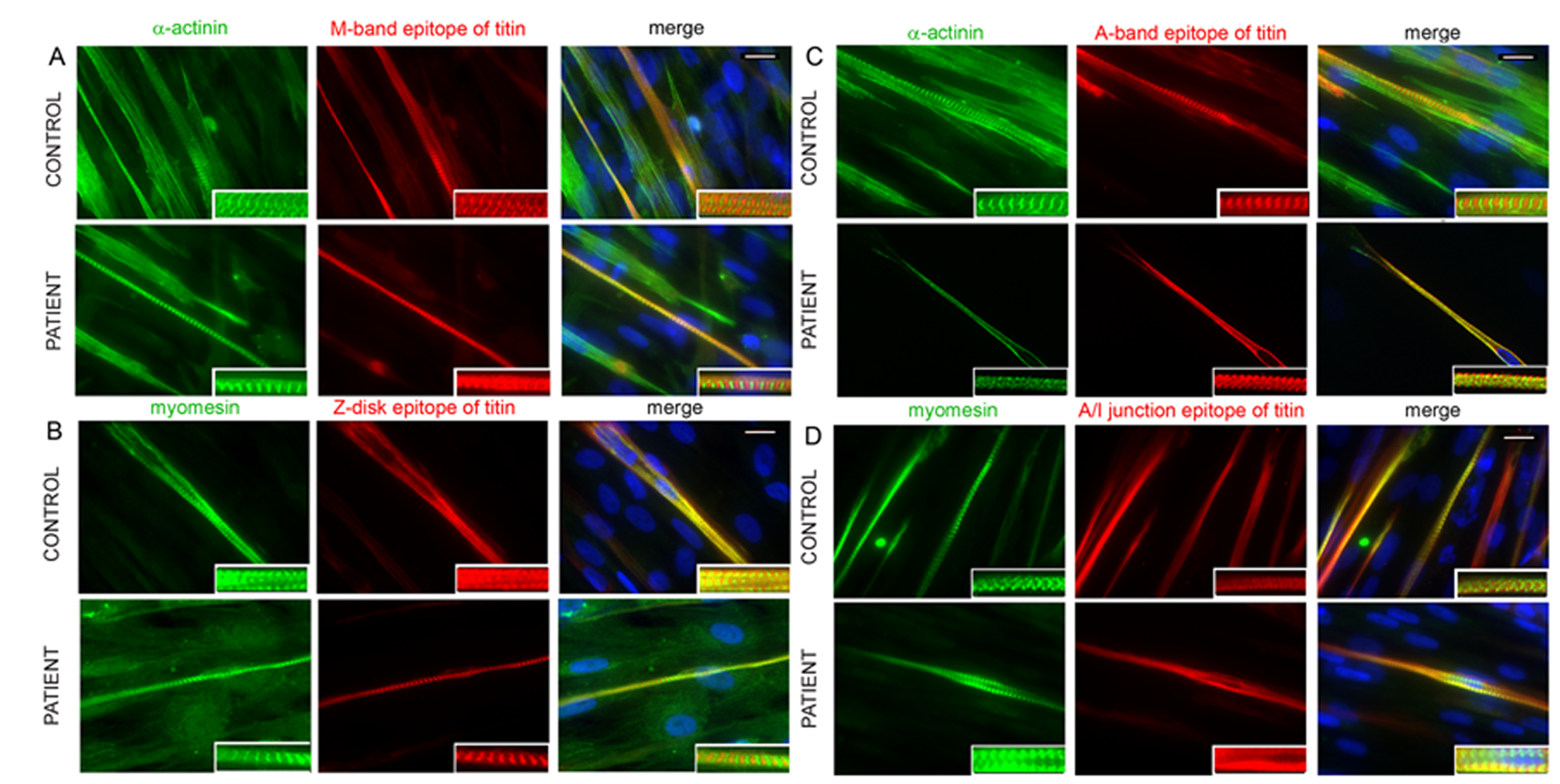

Supplement: S7 Fig — (A) Double staining were performed with α-actinin (green) and M-band epitope of titin (red). (B) myomesin (green) and Z-disc epitope of titin. (C) α-actinin (green) and A-band epitope of titin. (D) myomesin (green) and A/I junction epitope of titin (red). The results were visualized in Zeiss Axio Observer microscope (Carl Zeiss AG, Germany) at 63x magnification. All nuclei were counterstained with DAPI (blue). The Z-disc can be seen clearly with α-actinin and Z-disc epitope of titin, the M-band can be seen with myomesin and M-band epitope of titin and A-band can be seen with A-band and A/I junction epitope of titin in the patient and a control 6-day differentiated myotubes (insets). Note the strikingly thinner myotubes in the patient compared to the cells in the control. The bars represent 10 μm. (TIF) [file pone.0142094.s007.tif]
