# Supplementary material for: Developmental MYH3 Myopathy Associated with Expression of Mutant Protein and Reduced Expression Levels of Embryonic MyHC
Source: PLoS One. 2015 Nov 6;10(11):e0142094. doi: 10.1371/journal.pone.0142094 (PMC4636365; doi:10.1371/journal.pone.0142094)
Supplement: S1 File — (DOCX) [file pone.0142094.s008.docx]

**Supporting Information**

1. **Expanded Materials and Methods**
2. **Results**
3. **References**

**1. Expanded Materials and Methods**

**1.1 Culture of myoblasts and differentiation to myotubes**

Skeletal muscle cells from donors were enzymatically isolated and cultured, as previously described [[1](#_ENREF_1),[2](#_ENREF_2)]. All reagents were purchased from Invitrogen Life Technologies (Invitrogen, Carlsbad, CA, USA) unless otherwise specified.

**1.2 Isolation of RNA and protein from cultured cells**

Concurrent isolation of RNA and proteins from myoblasts and differentiated myotubes (D1-49) of the patient and five controls were performed using the RNeasy Plus Mini Kit (Qiagen, Hilden, Germany) following the manufacturer’s instruction. Briefly, after RNA was recovered from the cell lysate by isopropanol precipitation, proteins, which remained soluble in the isopropanol solution, were recovered by acetone precipitation. The isolated RNA was subjected for complementary DNA (cDNA) synthesis, polymerase chain reaction (PCR) amplifications, sequencing, restriction fragment length polymorphism (RFLP) and MyHC transcripts quantification analyses. The protein samples were subjected for immunoblotting.

**1.3 Complementary DNA (cDNA) analysis**

cDNA was synthesis from total RNA, using iScript cDNA Synthesis kit (Bio-Rad Laboratories, Sweden), according to the manufacturer’s instructions. To determine the appearance of the *MYH3* c.602C>T mutation in the cultured cells of the patient, a fragment of cDNA was amplified in a master mixture (ReddyMix PCR Master Mix, ABgene House, Blenheim Road, UK). The polymerase chain reaction (PCR) amplifications consisted of an initial denaturing step for 3 minutes at 94°C, followed by denaturation at 94°C for 1 minutes, annealing at 57°C for 1 minutes, extension at 72°C for 1 minutes for 35 cycles and a final extension step of 72°C for 10 minutes. The primer pairs 5’-TGATCGTGAAAACCAGTCCATTCT-3’ (corresponding to nucleotide 581-604 of human *MYH3*, GenBank accession number: NM_002470.2) combined with 5’-TTGGCCAGGTCCCCAGTAGCT-3’ (corresponding to nucleotide 697-677) cover cDNA sequences flanking exon 4 through exon 5 of *MYH3*. The resulting PCR products were analysed by sequencing. The nucleotide sequence determination was carried out by cycle sequencing using an ABI 3730xl DNA sequencer (GATC Biotech AG, Konstanz, Germany) and analysed using the NCBI BLAST website (<http://www.ncbi.nlm.nih.gov/BLAST/>).

For RFLP analysis of the *MYH3* c.602C>T mutation in cDNA, a 308-bp fragment was amplified using the primer pairs 5’-ACCGTTACACATCTTGGATG-3’ combined with 5’-GGAGTCCTTCTTCTTGGCCA-3’. The removal of a *BsaWI* restriction enzyme site by the c.602C>T mutation was determined by digesting the resulting PCR fragment with the *BsaWI* (New Englands Biolabs, Beverly, MA).

**1.4 Quantitative analysis of mutant and wild-type MYH3 transcripts**

The analysis of the proportion of mutated p.Thr178Ile and wild-type *MYH3* transcripts was performed as described in supplementary material for RFLP analysis, apart from the use of the fluorescein-labeled (6-carboxyfluorescein (FAM)) forward primer. After restriction enzyme cleavage of the amplified 308-bp fragment with *BsaWI*, the digested fragment was separated in polyacrylamide gel. The relative amount of the wild-type and mutated embryonic MyHC transcripts were then calculated from the respective peak areas of cleaved and uncleaved transcripts.

**1.5 Quantitative analysis of MyHC transcripts in the differentiated myotubes**

PCR was performed on cDNA from myoblasts (D0) and differentiated myotubes (D1, 3, 6, 8, 10, 16, 24, 32 and 49) with the fluorescein-labeled primers to analyse the percentage of the three major MyHC isoforms, MyHC I, MyHC IIa and MyHC IIx, in addition to the embryonic and perinatal MyHCs, as previously described ([12](#_ENREF_12)). Briefly, amplification of MyHC IIa, MyHC IIx and embryonic and perinatal MyHCs using a fluorescein-labeled (6-carboxyfluorescein (FAM)) forward primer results in 505-, 499-, and 496-bp fragments, respectively. Amplification of MyHC I, using a MyHC I-specific fluorescein-labeled (hexachlorocarboxyfluorescein (HEX)) forward primer results in a 496-bp fragment. In addition, PCR was performed on cDNA to discriminate the expression of embryonic and perinatal MyHC. A fluorescein-labeled (6-carboxyfluorescein (FAM)) forward primer; 5’-TCTTGGAAAGTCCAACAA-3’ (corresponding to nucleotide 1759-1776) combined with the backward primer; 5’-CAAACGTGGCATAGAGGTG-3’ (corresponding to nucleotide 1946-1964) results 205-bp embryonic MyHC fragment. A fluorescein-labeled (6-carboxyfluorescein (HEX)) forward primer; 5’-CCTGGGCAAGTCTGCCAA-3’ (corresponding to nucleotide 1772-1790 of human *MYH8*, GenBank accession number: NM_002472) combined with the backward primer; 5’-CATACGTGGAAAAGAGACT-3’ (corresponding to nucleotide 1958-1977 of human *MYH8*) results 205-bp perinatal MyHC fragment.

**1.6 Real-time RT-PCR**

Quantitative determination of embryonic MyHC isoform mRNA levels was performed by real-time RT-PCR (TaqMan Gene Expression Assays; Life Technologies, Carlsbad, CA, USA), using primer pairs amplifying *MYH3* (Hs01074230_m1) and specific Taqman probes. Glyceraldehyde-3-phosphate dehydrogenase (GAPDH) was used as endogenous control. qPCR reactions were performed in 20 μl mixtures containing 24 ng mRNA, 900 nM of each primer, and 200 nM probe. The *MYH3* copy number was determined by the *MYH3*/GAPDH ratio. The *MYH3* transcript level of the cultured, differentiated myotubes (D3, D6, D8, D10 and D15) from the patient was then compared to five controls.

**1.7 Gel electrophoresis and immunoblotting of cultured cells**

After heating to 100 °C for two minutes in Laemmli Buffer (Bio-Rad Laboratories, Hercules, CA) extracted protein samples from either myoblasts or differentiated cells were separated by 8% sodium dodecyl sulphate polyacrylamide gel electrophoresis (SDS-PAGE). Separated proteins were transferred to polyvinylidene difluoride membranes and blots were probed with either the primary mouse monoclonal anti IgG embryonic MyHC antibody (1:100, NovoCastra^TM^Lyophilized), rabbit anti MuRF1 antibody (1:50, Santa Cruz) or mouse monoclonal anti IgG perinatal MyHC antibody (NCL-MHCn) (1:25, NovoCastra^TM^Lyophilized). After incubation with either mouse or rabbit horseradish peroxidase (HRP)-conjugated secondary goat IgG polyclonal antibodies (1:2500; Pierce Biotechnology Inc., Rockford, IL, USA) the immunoreaction was detected with SuperSignal West Femto chemiluminescent substrate (Pierce), captured with a Fujifilm LAS 4000 CCD camera, and analyzed with Multi Gauge v3.1 software. The expression level of α-tubulin was used as a loading control. After WB analysis of embryonic MyHC or MuRF1, the membrane was stripped and incubated overnight at 4°C with a monoclonal mouse anti-α-tubulin antibody, washed 3 × 5 min in wash buffer, and incubated for 1h with HRP-conjugated secondary goat anti-mouse antibody (1:2,500; Pierce Biotechnology Inc., Rockford, IL, USA). The signal was detected and analysed as described above.

- 1. **Sample preparation for proteomic analyses**

Cultured cell pellet were lysed in lysis buffer (8 M urea, 4 % 3-[(3-cholamidopropyl)dimethylammonio]-1-propanesulfonate, CHAPS, 0.2 % sodium dodecylsulphate, SDS, 5 mM EDTA, 50 mM triethylammoniumbicarbonate (TEAB)) and protein concentrations measured using the Pierce 660 nm protein assay (Pierce Biotechnology Inc., Rockford, IL, USA). Samples were digested into peptides using the filter-aided sample preparation (FASP) method with deoxycholate addition to the digestion buffer essentially as described (Leon IR et al, 2013, Molecular and Cellular Proteomics, 12:2992-3005). Briefly, samples were diluted four-fold in 8 M urea and applied to 30 K molecular-weight cut-off spin filters (Pall NanoSep, Sigma-Aldrich, St. Louis, MO, USA), before washing on the membrane (8 M urea). Reduction (5 mM Tris-2-carboxyethyl phosphine, TCEP), alkylation (10 mM *S*-methyl methanethiosulfonate, MMTS) and tryptic digestion were each carried out in 100 uL digestion buffer (1 % sodium deoxycholate, Sigma-Aldrich, with 20 mM TEAB) with centrifugation steps to remove reduction and alkylation solution in turn. Tryptic digestion was performed by a double-digestion strategy with two separate additions of sequencing grade porcine trypsin (Promega Corp., Madison, WI, USA, 1:100 trypsin:protein ratio).

Tryptic peptides were collected by centrifugation into Axygen Maxymum Recovery vials (VWR International, Stockholm, Sweden). The deoxycholate detergent was precipitated by acidification adding formic acid to a final concentration of 2 % followed by centrifugation at 13 000 rpm for 10 minutes. The supernatant was collected and peptides were fractionated into five fractions by strong cation-exchange chromatography using spin columns (Pierce Strong Cation Exchange spin columns, Pierce Biotechnology Inc., Rockford, IL, USA). Fractions were generated by elution using increasing B-buffer content (5, 10, 20, 40 and 100%). Buffers A and B constituted 5 and 500 mM ammoniumformate, pH 2.8, in 25 % acetonitrile, respectively. Fractions were evaporated to dryness and desalted using Pierce C18 spin columns (Pierce Biotechnology Inc., Rockford, IL, USA) and reconstituted to in 15 µL 3 % acetonitrile with 0.1 % formic acid prior to LC-MS analysis.

**1.9 Targeted LC-MS/MS analysis**

Initially, a data-dependent acquisition and database search strategy identified the peptide tryptic peptide ENQSILITGESGAGK from human myosin-3 in the sample (see supplementary material section 1.12). To test for the possible presence of the corresponding mutated tryptic peptide ENQSILIIGESGAGK, a targeted data acquisition strategy was employed using a Q Exactive instrument as described below. Importantly, the ENQSILIIGESGAGK peptide was determined to be unique in the human proteome by BLAST searching all possible sequence combinations against the NCBI non-redundant database (i.e. all possible combinations of I/L substitutions as these amino acid residues are isobaric).

Data was acquired using a Q Exactive - Easy-nLC 1000 instrument combination (both Thermo Fisher Scientific, Inc., Waltham, MA, USA). Peptides (3 µL injection volume) were separated using an in-house constructed pre-column and analytical column set up (45x0.075 mm I.D and 200x0.750 mm I.D., respectively) packed with 1.8 μm Reprosil-Saphir C18 particles (Dr. Maisch GmbH, Ammerbuch, Germany). The following gradient was run at 200 nL/min; 7-27 % B-solvent (acetonitrile in 0.2% formic acid) over 70 min, 27-40 % B over 10 min, 40-80 % B over 5 min with a final hold at 90 % B for 10 min.

The Q Exactive mass spectrometer was set to continuously acquire two types of data in positive ion mode for the duration of the gradient, both at a resolution of 140 000 (FWHM at *m/z* 200). Full MS spectra were acquired over the *m/z* range 400-800 including an internal calibrant ion at *m/z* 445.120023. Further, fragmentation data was collected for the theoretical *m/z* values of the doubly charged peptide ions of interest, ENQSILITGESGAGK (*m/z* 752.38610) and ENQSILIIGESGAGK (*m/z* 758.40429). Parameters were set for multiplexed MS/MS data collection using the data-independent acquisition (DIA) function of the Q Exactive control software (version 2.2). For both MS and MS/MS spectra, the automated gain control (AGC) threshold was set to 3e6 with a maximal injection time of 500 msec (for DIA fragmentation, the injection time is split equally between the two precursors multiplexed in each MS/MS OrbiTrap scan). Fragmentation data was collected for an isolation width of 1.2 Da at a normalized collision energy of 30.

- 1. **Targeted data analysis**

Analysis was performed using the SkyLine software package (version 2.1, developed by the MacCoss lab, University of Washington).

**1.11 LC-MS/MS Analysis on LTQ-Orbitrap Velos**

Fractions (2 µL injections) were analyzed on a LTC-OrbiTrap Velos - Easy-nLC II instrument combination (both Thermo Fisher Scientific, Inc., Waltham, MA, USA). Peptides (3 µL injection volume) were separated using an in-house constructed pre-column and analytical column set up (45x0.075 mm I.D and 230x0.750 mm I.D., respectively) packed with 3 μm Reprosil-Pur C18-AQ particles (Dr. Maisch GmbH, Ammerbuch, Germany). The following gradient was run at 200 nL/min; 5-35 % B-solvent (acetonitrile in 0.2% formic acid) over 74 min, 35-50 % B over 10 min, 50-80 % B over 2 min with a final hold at 80 % B over 4 min.

Ions were injected into the LTQ-Orbitrap Velos mass spectrometer under a spray voltage of 1.6 kV in positive ion mode. For MS scans, 1 microscan was acquired at 30 000 resolution (at *m/z* 400) over a mass range of *m/z* 400-1400. MS analysis was performed in a data-dependent mode, with the top 10 most abundant doubly or multiply charged precursor ions in each MS scan selected for fragmentation (MS^2^) by collision-induced dissociation (CID). For MS^2^ scans, 1 microscan was collected with an isolation width of 2 at a normalized collision energy of 35 for an activation time of 10 msec.

**1.12 Database Search**

MS raw data files from fractions were merged for identification using Proteome Discoverer version 1.3 (Thermo Fisher Scientific, Inc., Waltham, MA, USA). A database search of the UniProtKB/Swissprot database (Swiss Institute of Bioinformatics, Switzerland, downloaded September 2013) restricted to the human taxon was performed using the Mascot search engine (version 2.3, Matrix Science LTD., London, United Kingdom). Precursor ion tolerance was 10 ppm with a fragment ion tolerance of 500 molecular mass units. Tryptic peptides were accepted with 1 missed cleavage and variable modifications of methionine oxidation and cysteine methylthiolation. Confidence of the peptide identification step was estimated using a target-decoy database search strategy limited to a false-discovery rate (FDR) of 1 %. The cut-off point corresponding to a 1 % FDR was at a Mascot confidence ≥99.43 %. Peptides above this threshold were included for protein grouping identifying a total of 1037 protein groups with a minimum of two valid peptides per protein.

**1.13 Fusion index assessment**

The fusion index was calculated as published before [[3](#_ENREF_3)]. Briefly, the number of nuclei in multinucleated myotubes divided by the total number of nuclei in cultures at day 3 and day 6 of *in vitro* differentiation, where at least 200 nuclei were counted. Nucleation size histogram represents detailed analysis of nucleation size frequency of 2-3 nucleated, 3-10 nucleated and 10-30 nucleated myotubes at day 3 and day 6 of *in vitro* differentiation.

**1.14 Immunofluorescence analysis of cultured myotubes and microscopy**

At given time points, myotubes plated on chamber slides were washed three times in phosphate-buffered saline (PBS) and fixed with 4% formaldehyde for 10 min. Free aldehyde groups were blocked with 50mM NH_4_Cl for 10 min and cells were permeabilised in PBS containing 0.1% Triton X-100 for 4 min. Cells were blocked with 1% bovine serum albumin (BSA) in PBS for 30 min prior to staining. The antibodies used in the study were: anti-Z-disc titin (T12) [[4](#_ENREF_4)], anti-A/I-junction titin (T3) [[4](#_ENREF_4)], anti-A-band titin (T30) [[5](#_ENREF_5)] and anti-M-band titin (T51) [[6](#_ENREF_6)], anti-myomesin BB75 [[6](#_ENREF_6)], anti-M-protein (AA259), anti-α-actinin (653), anti-myosin binding protein C (BB88), anti-embryonic MyHC (NCL-MHCd), anti-perinatal MyHC (NCL-MHCn) (NovoCastra^TM^Lyophilized) and anti-MyHCs (developmental studies hybridoma bank, Iowa, USA).

Secondary antibodies goat anti either mouse or rabbit IgG conjugated to Alexa Fluor-488, Alexa Fluor-564, or Alexa Fluor-647 were purchased from Molecular Probes, NY, USA, goat anti-mouse IgA conjugated to FITC was purchased from Southern Biotech, Birmingham, USA, and goat anti-rabbit IgG conjugated to TexasRed was purchased from DAKO Cytomation, Glostrup, DA. Filament actin was visualised using Alexa Fluor® 555-conjugated phalloidin obtained from Molecular Probes. Stained cells were mounted in Prolong® Gold antifade reagent with DAPI to highlight cell nuclei and imaged using a Zeiss Axio Observer microscope using a 40x or 63x oil objective. Images were processed using Photoshop software (Adobe, USA).

**2. Results**

Double-immunostaining for either α-actinin, M-band epitope of titin or Z-disc epitope of titin and myomesin demonstrated the expected alternating pattern of Z-disc and M-band in patient myotubes (S7A and S7B Fig). In addition, double-immunostaining for α-actinin and A-band epitope of titin or myomesin and A/I junction epitope of titin demonstrated well-ordered appearance of sarcomere (S7C and S7D Fig).

**3. References**

1. Vilquin JT, Marolleau JP, Sacconi S, Garcin I, Lacassagne MN, et al. (2005) Normal growth and regenerating ability of myoblasts from unaffected muscles of facioscapulohumeral muscular dystrophy patients. Gene Ther 12: 1651-1662.

2. Lecourt S, Marolleau JP, Fromigue O, Vauchez K, Andriamanalijaona R, et al. (2010) Characterization of distinct mesenchymal-like cell populations from human skeletal muscle in situ and in vitro. Exp Cell Res 316: 2513-2526.

3. Bello NF, Lamsoul I, Heuze ML, Metais A, Moreaux G, et al. (2009) The E3 ubiquitin ligase specificity subunit ASB2beta is a novel regulator of muscle differentiation that targets filamin B to proteasomal degradation. Cell Death Differ 16: 921-932.

4. Furst DO, Osborn M, Nave R, Weber K (1988) The organization of titin filaments in the half-sarcomere revealed by monoclonal antibodies in immunoelectron microscopy: a map of ten nonrepetitive epitopes starting at the Z line extends close to the M line. J Cell Biol 106: 1563-1572.

5. Furst DO, Osborn M, Weber K (1989) Myogenesis in the mouse embryo: differential onset of expression of myogenic proteins and the involvement of titin in myofibril assembly. J Cell Biol 109: 517-527.

6. Obermann WM, Gautel M, Steiner F, van der Ven PF, Weber K, et al. (1996) The structure of the sarcomeric M band: localization of defined domains of myomesin, M-protein, and the 250-kD carboxy-terminal region of titin by immunoelectron microscopy. J Cell Biol 134: 1441-1453.
